# In Situ Development of Quercetin-Enhanced Layered Double Hydroxides for Targeted Osteosarcoma Therapy

**DOI:** 10.1021/acsbiomaterials.5c01290

**Published:** 2025-11-21

**Authors:** Panagiota Zygouri, Grigorios Tsiodoulos, Marilena Lianou, Antrea-Maria Athinodorou, Eirini Papanikolaou, Yannis V. Simos, Konstantinos Spyrou, Mohammed Subrati, Angela S. Kaloudi, Konstantinos Tsamis, Lampros Lakkas, Zili Sideratou, Fotios K. Katsaros, Dimitrios Peschos, Vasileios Ragos, Dimitrios P. Gournis

**Affiliations:** † Department of Materials Science and Engineering, University of Ioannina, 45110 Ioannina, Greece; ‡ Nanomedicine and Nanobiotechnology Research Group, University of Ioannina, 45110 Ioannina, Greece; § Department of Maxillofacial, Faculty of Medicine, School of Health Sciences, University of Ioannina, 45110 Ioannina, Greece; ∥ Department of Physiology, Faculty of Medicine, School of Health Sciences, University of Ioannina, 45110 Ioannina, Greece; ⊥ Institute of Nanoscience and Nanotechnology, NCSR “Demokritos”, Aghia Paraskevi, 15310 Attikis, Greece; # School of Chemical and Environmental Engineering, Technical University of Crete (TUC), GR-73100 Chania, Crete, Greece; ∇ Institute of GeoEnergy, Foundation for Research and Technology-Hellas, GR-73100 Chania, Crete, Greece

**Keywords:** layered double hydroxides, quercetin, intercalation, cytotoxicity, cancer

## Abstract

In this study, we investigated the structural, thermal, and cytotoxic properties of Mg–Al layered double hydroxides (LDHs) intercalated with quercetin. Fourier transform infrared (FTIR) and Raman spectroscopy confirmed the successful incorporation of quercetin into the LDH structure, while X-ray diffraction (XRD) showed an increased interlayer spacing due to quercetin intercalation. Thermogravimetric analysis (TGA) demonstrated greater thermal degradation in quercetin-intercalated LDHs compared to nitrate-intercalated LDHs, suggesting a higher quercetin content in the material. Atomic force microscopy (AFM) indicated morphological changes with quercetin, particularly larger surface clusters at room temperature. Cytotoxicity assays revealed that quercetin-intercalated LDHs exhibited a dose- and time-dependent reduction in cell viability, which was more pronounced at higher concentrations and longer exposure times. Quercetin alone had a stronger cytotoxic effect on NIH/3T3 cells compared with Saos-2 cells. Additionally, reactive oxygen species (ROS) assays showed distinct effects of quercetin on both cell lines. These findings highlight the potential of quercetin-LDH hybrids for biomedical applications, particularly for targeted drug delivery.

## Graphical abstract

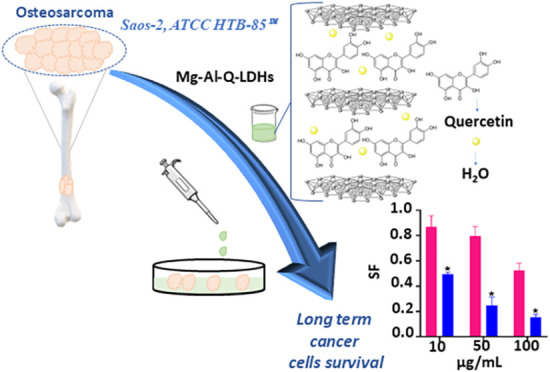

## 1 Introduction

Over the past years, inorganic materials such as gold nanoparticles (AuNPs), mesoporous silica nanoparticles, quantum dots (QDs), carbon nanotubes (CNTs), layered double hydroxides (LDHs), etc. have been widely studied as drug delivery vehicles.^1–5^ Numerous studies in the literature report that inorganic nanoparticles are nontoxic and more stable than organic materials. Additionally, the ability to control the release of pharmaceutical drugs makes inorganic nanoparticles ideal for use as drug carriers.^6–8^

LDHs are layered materials composed of positively charged sheets with a general type of [M_1–*x*_^2+^M_*x*_^3+^(OH)_2_] (A^*n*–^)_*x*/*n*_·mH_2_O of a bivalent metallic cation and trivalent metallic cation, while A^*n*‑^ is an interlayer anion placed in the interlamellar space of this layered nanomaterial to compensate for the positive charge.^9^ This unique structure of LDH places them as one of the most prominent nanocarrier groups. Along with the previously mentioned advantages of inorganic particles, LDH nanosheets have also gained the scientific community’s attention due to their high flexibility in chemical composition and pH-dependent solubility, particularly in the case of Mg/Al-LDHs, as anticancer drug nanocarriers. Due to the anion exchange properties of double hydroxide, a considerable number of substances have been intercalated into the interlayer of LDHs nanogalleries,^1,2,10^ including anti-inflammatory drugs (NSAIDs) such as ibuprofen, diclofenac, naproxen, fenbufen, ketoprofen, and indomethacin,^11^ anticancer drugs such as Methotrexate, 5-Fluorouracil, and Doxifluridine, and cardiovascular drugs such as captopril, heparin, pravastatin, and fluvastatin.^12^ The unique aspects of LDH lie in its anion exchange capacity, which is correlated to the M^+2^/M^+3^ ratio. This capacity allows for the loading of anionic drugs and defines drug density, while the low toxicity of LDH sheets from Mg and Al metals protects the drug from degradation. Finally, the biocompatibility of LDH allows slow release in acidic environments via dissolution of the layered material.^13^

Flavonoids belong to the class of phenolic compounds and are produced as secondary metabolites in plants.^14,15^ They consist of 15 carbon atoms, which are arranged in a C6–C3–C6 order, and two aromatic rings, A and B, linked by a three-carbon chain bridge.^15,16^ Flavonoids are found in abundance in plant-origin foods and beverages, such as fruits, vegetables, tea, and wine.^16,17^ These compounds have a positive impact on health and are used as essential ingredients in a variety of nutritional, pharmaceutical, and cosmetic products. Their large number of applications is due to their antioxidant, anti-inflammatory, antiviral, and anticancer properties, as well as their role in cellular enzyme regulation.^17,18^ Quercetin is a flavonoid belonging to the class of flavanols and is found in abundance in foods such as apples, onions, nuts, and tea. It is characterized by its yellow color and low aqueous solubility.^19^ Quercetin is beneficial for human health due to its antioxidant properties, which help prevent oxidative stress and the formation of reactive oxygen species. It also has antiviral and anti-inflammatory effects through the inhibition of inflammation-associated enzymes and signaling pathways.^19,20^

It is well established in the literature that LDHs possess an exceptionally high drug-loading capacity, surpassing most other 2D nanomaterials.^21^ This enables them to carry substantial amounts of active substances, such as quercetin. Moreover, LDHs can stabilize labile bioactive molecules and enhance the solubility of poorly soluble drugs, making them excellent candidates for delivering quercetin, which has an inherently low aqueous solubility.

LDHs have been widely employed to overcome the hydrophobicity of bioactive compounds. For instance, Murath et al. used LDHs as carriers for ellagic acid, a polyphenolic antioxidant with poor water solubility,^22^ while Kleyi et al. incorporated folic acid into LDH-based formulations for topical skincare delivery.^23^ In both cases, the molecules retained their antioxidant activity. Similarly, epigallocatechin-3-gallate (EGCG), another polyphenol, was intercalated into Ca/Al–NO_3_ LDHs, and its anticancer properties were successfully evaluated against a prostate cancer cell line (PC3).^24^

Osteosarcoma treatment typically involves a multimodal approach, including surgery, chemotherapy, and radiotherapy, with chemotherapy remaining the standard of care. However, its use is often limited by severe side effects. Consequently, there is growing interest in developing new strategies such as targeted therapies, immunotherapy, gene therapy, and advanced drug delivery systems, which represent promising avenues of ongoing research.^25^ As drug delivery systems, LDHs have been successfully employed in vitro to deliver siRNA (cell death–siRNA) to osteosarcoma U2OS cells^26^ and celastrol, a triterpenoid compound, to human osteosarcoma cell lines (143B and HOS). They have also been used as cGAMP carriers to enhance the infiltration of CD40^+^ macrophages and dendritic cells,^27^ and in vivo as methotrexate carriers for the treatment of osteosarcoma-induced Balb/c nude mice.

To the best of our knowledge, quercetin has not yet been intercalated into LDH nanosheets or systematically evaluated for its cytotoxic activity in vitro. Therefore, we aimed to investigate the *in vitro* cytotoxicity of newly synthesized Mg–Al–NO_3_-LDH nanosheets conjugated with quercetin, using both normal and cancer cell lines.^28^

## 2 Materials and Methods

### 2.1 Materials and Methods

Magnesium nitrate hexahydrate [Mg­(NO_3_)_2_·6H_2_O], aluminum nitrate nonahydrate [Al­(NO_3_)_3_·9H_2_O], sodium nitrate (NaNO_3_), and sodium hydroxide (NaOH, pellets) were purchased from Merck. Quercetin, an antioxidant flavonoid, was supplied by the Tokyo Chemical Industry (TCI).

Dulbecco’s modified Eagle’s medium (DMEM) high glucose, phosphate buffered saline (PBS), thiazolyl blue tetrazolium bromide (MTT), 2′,7′-dichlorofluorescin diacetate (DCFDA, ≥97%), and crystal violet were sourced from Sigma-Aldrich Chemical Co. (St. Louis, MO, USA). Fetal bovine serum (FBS) was procured from PAN BIOTECH (Aidenbach, Germany). Trypsin-EDTA, penicillin-streptomycin, and l-glutamine were obtained from Biowest (Riverside, USA). Hanks’ Balanced Salt Solution (HBSS) was acquired from Biosera (Nuaille, France). Propidium iodide (PI) was purchased from BioLegend (San Diego, CA, USA). Glutaraldehyde (25%) and dimethyl sulfoxide (DMSO) were acquired from Thermo Fisher Scientific Pharmaceutics, Inc. (Waltham, MA, USA).

### 2.2 Synthetic Procedure of Mg–Al–NO_3_-LDHs

Double-layered hydroxides, with inorganic anions NO_3_^–^ in their interlamellar space, were synthesized via the sol–gel method. More specifically, two solutions were prepared: in the first (A) 3.5 gr (0.175 mol) of sodium hydroxide NaOH and 0.85 gr (0.02 mol) of sodium nitrate NaNO_3_ were dispersed in 35 mL of distilled water, while in the second (B) 9.61 g of (0.075 mol) Mg­(NO_3_)_2_·6H_2_O and 4.69g of (0.025 mol) Al­(NO_3_)_3_·9H_2_O were added to 25 mL of distilled water. While stirring and heating to 70 °C in an oil bath of solution A, solution B was added dropwise for the sol–gel process to get started, i.e., the formation of aggregates and the change of color from clear to white. The stirring under heating lasted for 24 h, after which centrifugation (6000 rpm, 3 min) and 3 washes with distilled water were performed. Finally, drying was carried out in a furnace at 70 °C (Scheme 1a).

**Scheme 1:**
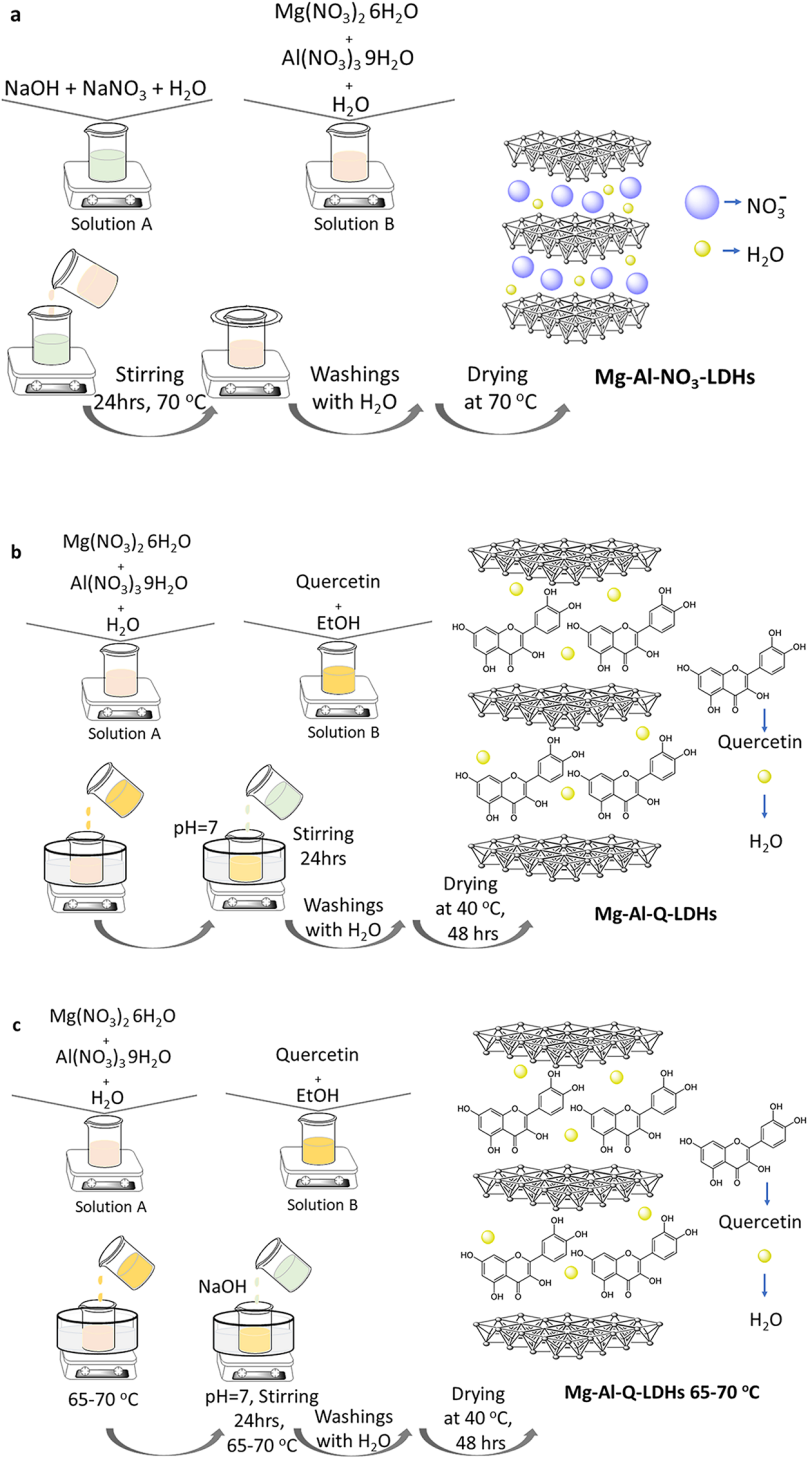
Schematic Representation of the Synthetic Procedure of (a) Mg–Al–NO_3_-LDHs, (b) Mg–Al-Q-LDHs, and (c) Mg–Al-Q-LDHs 65-70 °C

### 2.3 Synthetic Procedures of Mg-AI-Q-LDHs at Ambient Conditions and 65–70 °C

For the in situ synthesis of the Mg–Al-Q-LDHs, 2.4025 g of Mg­(NO_3_)_2_ 6H_2_O and 1.1725 g of Al­(NO_3_)_3_·9H_2_O were dispersed, under stirring, in a beaker in 10 mL of distilled water (Solution A). Then, in another glass, 0.285 g of quercetin was added to 4 mL of ethanol (Absolut) until this substance was completely dissolved (Solution B). Aqueous solution B was added dropwise to aqueous solution A while adjusting the pH value to 7 with sodium hydroxide solution (9.6 g NaOH and 30 mL H_2_O, 8M). The stirring lasted for 24 h. For the case of Mg-AI-Q-LDHs 65–70 °C, the aqueous solution B was added dropwise to the aqueous solution A and stirred in an oil bath at 65–70 °C, while adjusting the pH value to 7 with sodium hydroxide solution (9.6 g of NaOH and 30 mL of H_2_O, 8M). The stirring under heating at 65–70 °C lasted 24 h. Both synthesized materials were obtained after centrifugations (6000 rpm, 3 min) and 2 washings with H_2_O/EtOH (1:1 v/v). Finally, drying was carried out in a furnace at 40 °C for 48 h (Scheme 1b,c).

### 2.4 Loading of DAPI into Mg–Al-Q-LDHs 65–70 °C

DAPI (4′,6-diamidino-2-phenylindole) was loaded into Mg–Al-Q-LDHs 65–70 °C by mixing 10 mL of DAPI aqueous solution with a concentration of 0.5 mg/mL using sonication for 10 min. The mixture was stirred for 24 h at room temperature in the dark. The unreacted DAPI was removed by centrifugation at 13,000 rpm for 15 min. The process of centrifugation was repeated 6 times, each time washed with distilled water. The final material was collected after drying.

### 2.5 Cell Culture

In this study, fibroblasts from Albino Swiss mouse embryo (NIH/3T3, ATCC CRL-1658) and human osteosarcoma cell line (Saos-2, ATCC HTB-85) were used. Sterile 10 cm diameter dishes were used for growing the cells. Both cell lines were cultured using DMEM-high glucose, supplemented with penicillin-streptomycin (1%), l-glutamine (1%), and FBS (10%). Subcultures were done thrice a week, and the cells were preserved in 5% CO_2_ at 37 °C.

### 2.6 Determination of Cell Viability

Stock solutions of Mg–Al–NO_3_-LDHs (100 mg/mL), Mg–Al-Q-LDHs (100 mg/mL), Mg–Al-Q-LDHs 65–70 °C (100 mg/mL), and quercetin (20 mg/mL) were prepared in DMSO and stored in the dark at 4 °C. Fresh working solutions were prepared from the stock solutions for each experiment.

Cell viability was evaluated using 96-well plates. Each well was plated with 5000 cells (Saos-2 or NIH/3T3). Cells were exposed to increasing concentrations of 10–1000 μg/mL for Mg–Al–NO_3_-LDHs and Mg–Al-Q-LDHs 65–70 °C, 10–750 μg/mL for Mg–Al-Q-LDHs RT, and 1–200 μg/mL for quercetin. After an incubation period of 24 or 48 h, 40 μL of MTT (stock solution of 3 mg/mL) was introduced to each well. Following an additional incubation under the same conditions (37 °C and 5% CO_2_) for 3 h, the cell precipitate was dissolved in 100 μL of DMSO after the supernatant was removed. Absorbance at 540 and 690 nm was measured using a spectrophotometer (Infinite 200 Pro, Tecan, Switzerland).

### 2.7 Clonogenic Assay

Cells were plated in six-well plates at a density of 500 cells/mL with a final volume of 2 mL per well. Mg–Al–NO_3_-LDHs, Mg–Al-Q-LDHs, Mg–Al-Q-LDHs 65–70 °C, and quercetin were added after 24 h at concentrations of 1, 10, 50 μg/mL. After 24 h, the supernatant was removed, and fresh medium (2 mL) was added to each well. An 8-day incubation followed, with medium renewal on the fourth day. At the end of the incubation period, the medium was removed, and the cells were rinsed cautiously with PBS and stained for 30 min with 6.0% mixture of glutaraldehyde and 0.5% crystal violet. The dishes were carefully washed and dried at room temperature, the colonies were counted using the Open CFU open-source software (version 3.9.0),^29^ and the surviving fraction (SF) of the treated cells was calculated.^30^

### 2.8 Determination of Intracellular ROS Formation

A total of 75 × 10^3^ cells/mL were seeded in six-well plates, and 10 μg/mL Mg–Al–NO_3_-LDHs, Mg–Al-Q-LDHs, and Mg–Al-Q-LDHs 65–70 °C, or quercetin were added. Following a 24 h incubation, cells were washed with PBS, detached with trypsin, and placed in 2 mL of HBSS. The cells were then stained with DCFDA (2.5 μM) for 30 min at 37 °C in the dark. After the samples were stained, 1 μg/mL PI solution was added. The samples were put on ice and immediately analyzed using a fluorescence-activated cell sorting flow cytometer (Partec ML, Partec GmbH, Germany). Each sample involved the measurement of 10,000 events, and all experiments were replicated three times.

### 2.9 Characterization Techniques for Layered Double Hydroxides (LDHs)

FTIR spectra over the spectral range 400–4000 cm^–1^ were collected with a PerkinElmer Spectrum GX infrared spectrometer featuring a deuterated triglycine sulfate (DTGS) detector. Every spectrum was the average of 64 scans taken with a 2 cm^–1^ resolution. Samples were prepared as KBr pellets with ca. 2 wt % of sample. Raman spectra were recorded with a Micro-Raman system RM 1000 RENISHAW, using a laser excitation line at 532 nm (Nd–YAG), in the range of 1000–3500 cm^–1^. A power of 1 mW was utilized with a 1 μm focusing spot to avoid photodecomposition of the samples. XRD patterns were collected on a D8 Advance Bruker diffractometer by using Cu Kα radiation (40 kV, 40 mA) and a secondary beam graphite monochromator. The patterns were recorded in the 2-theta (2Θ) range from 2 to 80°, in steps of 0.02° and a counting time of 2 s per step. Small-angle and wide-angle X-ray scattering (SAXS-WAXS) diffractograms were acquired utilizing a Nano-inXider from Xenocs SAS (Grenoble, France), equipped with a Xenocs GeniX3D Cu Kα source (1.54189 Å wavelength) and two 2-D Pilatus X-ray detectors configured for concurrent SAXS-WAXS studies without a beam-stop. The samples were placed in stainless steel washers (0.5 mm in height) and secured with Kapton tape. The measurement time was established at 3600 s with a medium resolution. The q-range was documented from 2.60 × 10^–3^ to 4.20 Å^–1^. The integrated XSACT software was utilized for background subtraction and 1D merging of the spectra. Thermogravimetric measurements were carried out with a PerkinElmer Pyris Diamond TG/DTA. Samples of about 5 mg were heated in air from 25 to 900 °C at a rate of 5 °C/min. Atomic force microscopy (AFM) images were recorded on silicon wafer substrates using tapping mode with a Bruker Multimode 3D Nanoscope (Ted Pella, Inc., Redding, CA, USA).

### 2.10 Fluorescence Microscopy

Saos-2 cells were cultured on coverslips and incubated with DAPI-labeled nanoparticles (0.5 or 1.0 μg/mL) for 24 h, followed by fixation with 3.7% PFA. Images were captured using a Nikon Eclipse TS2-FL microscope with diascopic and epi-fluorescence illumination and analyzed with Nikon NIS-Elements software.

### 2.11 Statistical Analysis

The data are presented in the form of mean and standard deviation. Statistical analysis was conducted using the Student *t* test to determine the significant difference between the means of the data. A value of *P* < 0.05 indicated statistical significance, and the analysis was performed using the SPSS version 20.0 software. Figures were created using GraphPad Prism 8 software.

## 3 Results and Discussion

### 3.1 Structural and Morphological Characterization of Mg–Al–NO_3_-LDHs, Mg–Al-Q-LDHs, and Mg–Al-Q-LDHs 65–70 °C

The FTIR spectra of Mg–Al–NO_3_-LDHs, Mg-AI-Q-LDHs, and Mg-AI-Q-LDHs 65–70 °C are presented in Figure 1. In the spectrum of Mg–Al–NO_3_-LDHs, three bands are observed at 553, 672, and 781 cm^–1^, which correspond to the vibrations of Al–O and Mg–O bonds. The band at 1368 cm^–1^ is attributed to the asymmetric stretching vibrations of the nitrates (NO_3_^–^). Subsequently, the peak at 1624 cm^–1^ is assigned to bending vibrations of the water molecules, which are located in the interlayer space of the LDHs, while the broad peak at 3460 cm^–1^ is attributed to the stretching vibrations of the hydroxyl groups of the LDHs’ sheets. Studying the spectra of Mg–Al-Q-LDHs and Mg–Al-Q-LDHs 65–70 °C, the appearance of the two peaks in the region of 2800–3000 cm^–1^ is attributed to the stretching vibrations of the C–H groups of the quercetin molecules. The same peaks are detected in the spectrum of quercetin, which leads to the conclusion that the phenolic groups were successfully inserted into the sheets of LDHs. In the region of 500–1700 cm^–1^, new bands are observed, which come from quercetin, but compared to its original spectrum, they appear reduced and shifted, which is due to the overlapping between the material and quercetin.^31,32^

**Figure 1:**
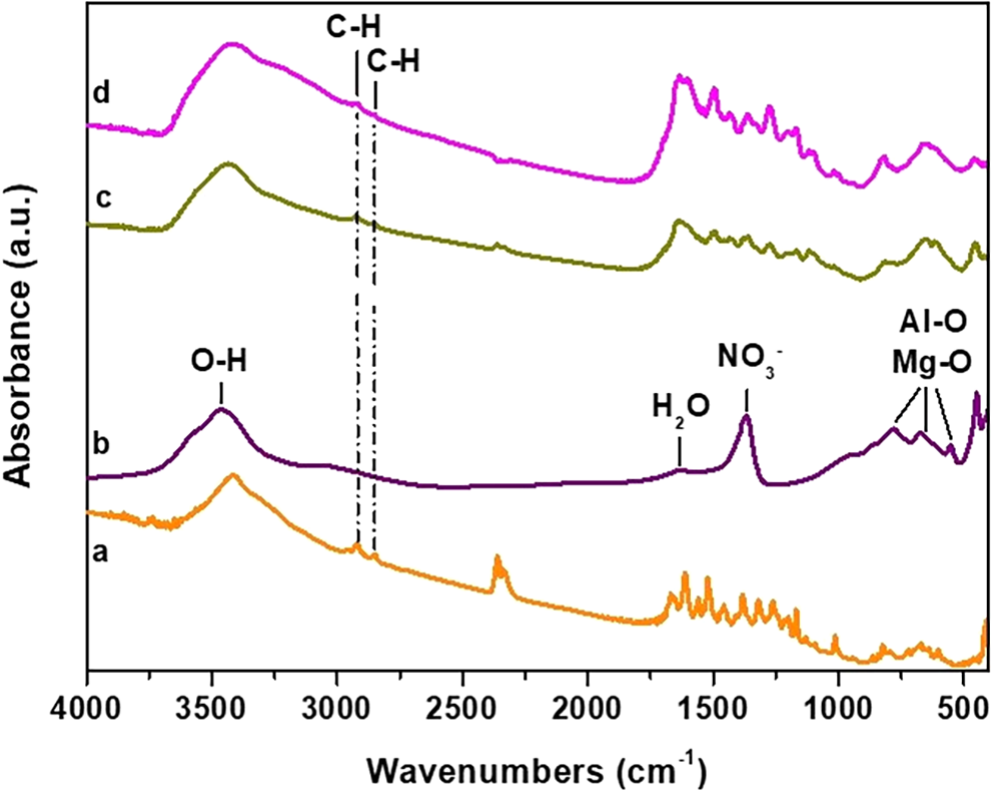
FTIR spectra of (a) Q, (b) Mg–Al–NO_3_-LDHs, (c) Mg–Al-Q-LDHs, and (d) Mg–Al-Q-LDHs 65–70 °C.

Figure 2 shows the Raman spectra of Mg–Al–NO_3_ Mg–Al-Q-LDHs, and Mg–Al-Q-LDHs 65–70 °C. The Raman spectrum of Mg–Al–NO_3_ LDH reveals an intense two-peak profile band in the 1000–1100 cm^–1^ region, and another weaker one at 555 cm^–1^. The intense band is typical of anion-intercalated LDHs irrespective of the type of anion, whereas the weaker band corresponds to the signature octahedral structure of brucite-type Al–O–Mg layers.^33^ The Raman spectra of Mg–Al-Q-LDHs and Mg–Al-Q-LDHs 65–70 °C confirm the successful intercalation of quercetin, which is evidenced by the (i) absence of the intense anion band in the 1000–1100 cm^–1^ region; (ii) emergence of the intense broad bands in the 1250–1700 cm^–1^ region, and the weaker ones at 860 and 1182 cm^–1^ characteristic of quercetin;^34^ and (iii) 34 cm^–1^ redshift (downshift) of the 555 cm^–1^ band.

**Figure 2:**
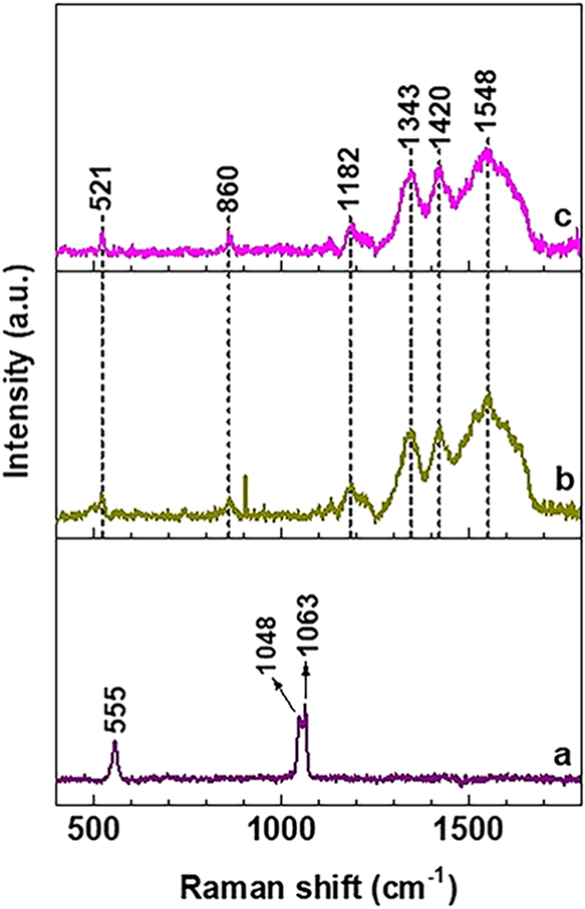
Raman spectra of (a) Mg–Al–NO_3_-LDHs, (b) Mg–Al-Q-LDHs, and (c) Mg–Al-Q-LDHs 65–70 °.

Figure 3 depicts the X-ray diffraction patterns of quercetin and Mg–Al–NO_3_-LDHs, Mg–Al-Q-LDHs, and Mg–Al-Q-LDHs 65–70 °C. The sample of Mg–Al–NO_3_-LDHs exhibits the main (003) basal reflex at 2θ = 11.55°, which, according to Bragg’s law, corresponds to the “basal spacing” of d_003_ = 7.7 Å (0.77 nm) characteristic of nitrate-intercalated LDHs. The “interlamellar spacing” is *c*_0_ = 0.77–0.478 = 0.292 nm. Taking into account the (110) reflex, the distance d_110_, at the particular angle 2θ, and the lattice parameter can be calculated. In the synthesized LDHs with quercetin without heating and at 65–70 °C, the main (003) basal reflex appears to be broader, with lower intensity and shifted from 11.55 to 10.02° (8.85 Å, *c*_0_ = 0.885–0.478 = 0.407 nm) and 9.65° (8.85 Å, *c*_0_ = 0.917–0.478 = 0.439 nm), respectively. This can be attributed to the increase of the interlamellar spacing due to the presence of quercetin molecules at the edges of the LDHs sheets, which distorts their lattice periodicity along the crystallographic *c*-axis.

**Figure 3:**
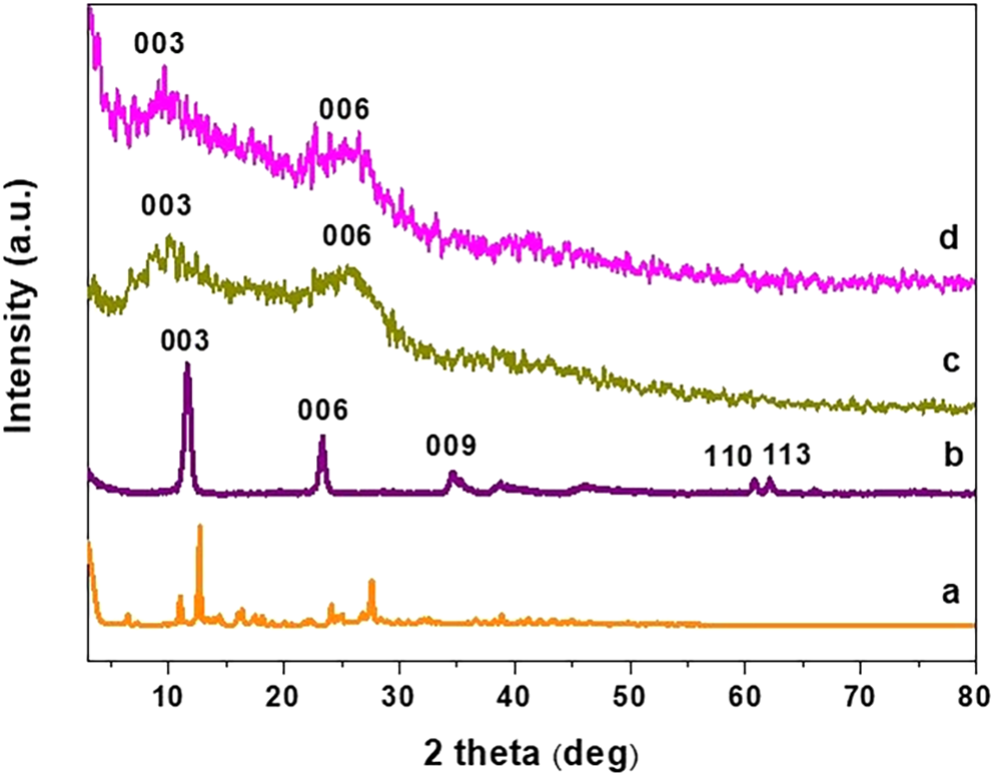
XRD patterns of (a) Q, (b) Mg–Al–NO_3_-LDHs, (c) Mg–Al-Q-LDHs, and (d) Mg–Al-Q-LDHs 65–70°.

Figure 4 shows the log–log small-angle and wide-angle X-ray scattering (SAXS-WAXS) diffractograms of Mg–Al–NO_3_, Mg–Al-Q, and Mg–Al-Q (65–70 °C) LDHs. The diffractogram of Mg–Al–NO_3_ (Figure 4a) reveals its characteristic reflexes in the high-q regime (*q* > 6 nm^–1^), i.e., WAXS region, most notably, the (003) and (006) Gaussian–Lorentzian reflexes corresponding to its basal and interlamellar spacings (d_003_ and d_006_), respectively.^35^ Moreover, the absence of these reflexes and the presence of the broad Lorentzian reflex at 15–20 nm^–1^ in the diffractograms of Mg–Al-Q and Mg–Al-Q (65–70 °C) (Figure 4b,c) reveal the loss of the lamellar ordering along the crystallographic *c*-axis due to the intercalation of the neutrally charged quercetin molecules. This is also corroborated by the ∼1 nm increase in the threshold real-space dimension (*D*_threshold_), i.e., the approximate *D* value above which the scattering intensity in the SAXS *q*-regime (*q* ≤ 6 nm^–1^) becomes proportional to *D*, of Mg–Al-Q and Mg–Al-Q (60–75 °C) relative to that of Mg–Al–NO_3_. To reveal further insights into the structural and morphological characteristics of the samples, the SAXS intensity was modeled by splitting it into three additive terms (eqs 1, 2, 3)­

1 $$I_{\mathrm{SAXS}}\left(q\right)=I_{\mathrm{particles}}\left(q\right)+I_{\mathrm{lamellae}}\left(q\right)+\mathrm{SF}$$

**Figure 4:**
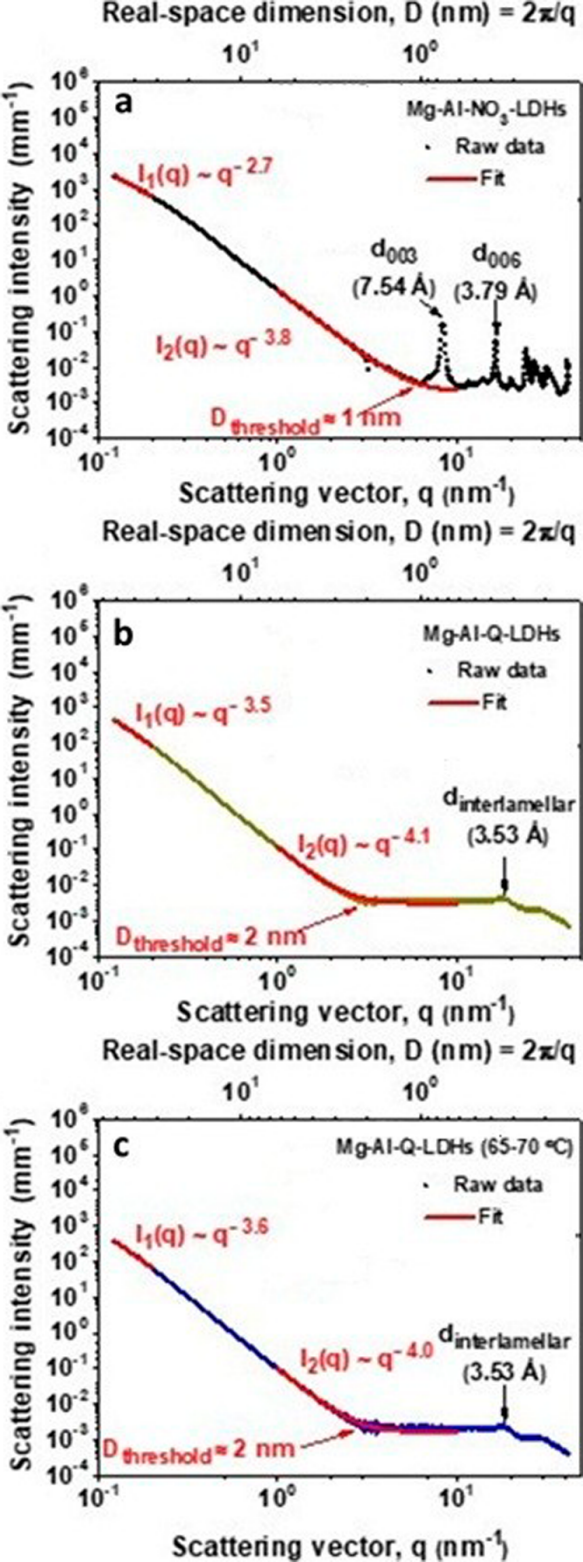
SAXS-WAXS analysis of (a) Mg–Al–NO_3_, (b) Mg–Al-Q, and (c) Mg–Al-Q (65–70 °C) LDHs. The fitting curve of the intermediate-q regime is extrapolated to 10 nm^–1^ to graphically demonstrate the horizontally asymptotic contribution of the structure factor.

The *I*_particles_(*q*) term corresponds to the dominant scattering contribution from the LDH particles and their clustered forms, and it can be determined by fitting the low-q regime (q → 0 nm^–1^) to Porod’s law:

2 $$\lim_{q\to 0}I_{\mathrm{SAXS}}\left(q\right)=I_{\mathrm{particles}}\left(q\right)=Aq^{-a}$$

The *I*_lamellae_(*q*) term contains the information about the interlamellar structure, whereas the *SF* term corresponds to the structure factor, which accounts for the ionic structure of the LDHs, and is assumed to be constant in the SAXS q-regime.^36^ Both terms, *I*_lamellae_(*q*) and *SF*, can be determined by fitting the intermediate-q regime (1–6 nm^–1^) as follows:

3 $$I\left(q\right),q\in \left(1,6\right)=I_{\mathrm{lamellae}}\left(q\right)=Bq^{-b}+C$$

where the *C* constant corresponds to the horizontally asymptotic SF term.^36^ It is worth noting that in eqs 2 and 3, both *A* and *B* are merely fitting parameters; however, the *a* and *b* exponents contain the sought-after information about the morphology and interlamellar environments of the LDHs, respectively. In the case of the Mg–Al–NO_3_ LDH, *a* = 2.7, whereas in the cases of the Mg–Al-Q and Mg–Al-Q (65–70 °C) LDHs, *a* > 3. This increase in the value of the *a* exponent can be attributed to the lower aspect ratios (aspect ratio = longest lateral dimension/thickness) of Mg–Al-Q and Mg–Al-Q (60–75 °C),^37^ thus confirming the successful intercalation of quercetin molecules into the interlamellar space of Mg–Al–NO_3_, which can be attributed to the increase in their aspect ratios, thus confirming the intercalation of quercetin molecules into their interlamellar spaces. The presence of the organic quercetin molecules between the inorganic lamellae of Mg–Al-Q and Mg–Al-Q (65–70 °C) LDHs is also evidenced by their higher values of the b exponent.^38,39^

Figure 5 shows the thermogravimetric analysis measurements of Mg–Al–NO_3_-LDHs, Mg–Al-Q-LDHs, and Mg–Al-Q-LDHs 65–70 °C. For comparison purposes, the quercetin curve is also presented. In the curve of Mg–Al–NO_3_-LDHs, 3 mass losses are observed. In more detail, up to 170 °C, the initial mass loss (6%) is due to the removal of the interlayer water molecules of the LDHs. Up to 275 °C, the second mass loss takes place, which is equal to 8% and is attributed to the dihydroxylation of the LDHs sheets. Finally, up to 550 °C, there follows a third weight loss (28%), a reduction due both to the completion of the dehydroxylation of the layers and to the removal of nitrate anions from the interlaminar space of the LDHs. The remaining percentage of the mass (42%) in these reactions corresponds to the metal oxides. In the thermodiagrams of Mg–Al-Q-LDHs and Mg–Al-Q-LDHs 65–70 °C, three mass losses are observed again, the first up to 170 °C due to the removal of water molecules and corresponds to a percentage of ∼8% for both materials. The second mass loss that takes place in the temperature range of 170–250 °C is attributed to the combustion of the organic molecules hosted on the surface and internal galleries of the LDHs’ layers, at a rate of ∼7% wt. Finally, the third mass loss at temperatures from 250 to 550 °C (76 and 73%, respectively) is due to the complete combustion of the rest of the organic moieties of the quercetin, as well as the dehydroxylation of the layers of LDH. Considering the above, it is concluded that in the in situ synthesis of LDHs with quercetin at ambient temperature, quercetin is introduced into the sheets of LDHs in greater proportions.

**Figure 5:**
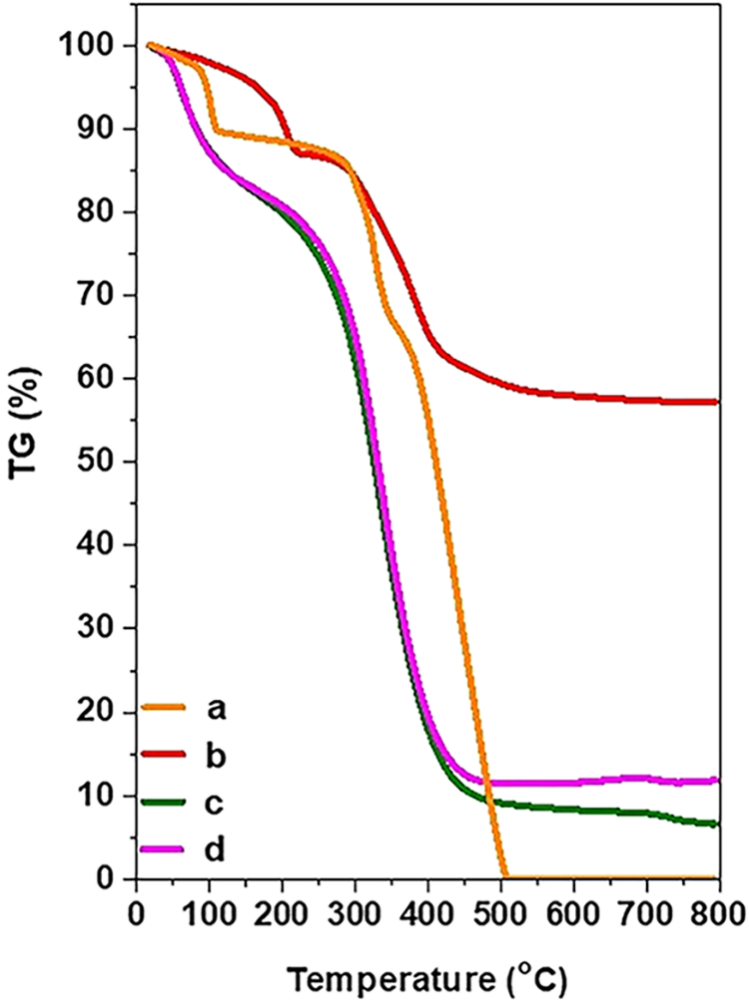
TGA thermographs of (a) Q, (b) Mg–Al–NO_3_-LDHs, (c) Mg–Al-Q-LDHs, and (d) Mg–Al-Q-LDHs 65–70 °C.

In Figure 6, representative AFM images of Mg–Al–NO_3_-LDHs, Mg–Al-Q-LDHs, and Mg–Al-Q-LDHs 65–70 °C are presented. The preparation of the samples included their deposition onto Si wafers from EtOH dispersions. The average thickness of the Mg–Al–NO_3_-LDHs was about 6 ± 1 nm (Figure 6a) in agreement with the literature.^40,41^ After the incorporation of quercetin in the interlayer space and on the surface of LDHs, the morphological characteristics differ compared to those of Mg–Al–NO_3_-LDHs. More specifically, in the case of Mg–Al-Q-LDHs at ambient conditions, the average thickness of the platelets was calculated at 8 ± 1 nm, while in the case of Mg–Al-Q-LDHs 65–70 °C, the average thickness was estimated at 4 ± 1 nm (Figure 6b). According to the section analysis of AFM images, in both the hybrid nanomaterials, the thickness of the LDH layers, including the quercetin incorporated in their interlamellar space, is 2 nm; however, the quercetin existing on the surface of the LDH is 6 nm for Mg–Al-Q-LDHs at room temperature and 2 nm for Mg–Al-Q-LDHs synthesized at 65–70 °C. This observation leads us to the conclusion that quercetin creates bigger clusters on the surface for the synthetic procedure happening without any heating treatment.

**Figure 6:**
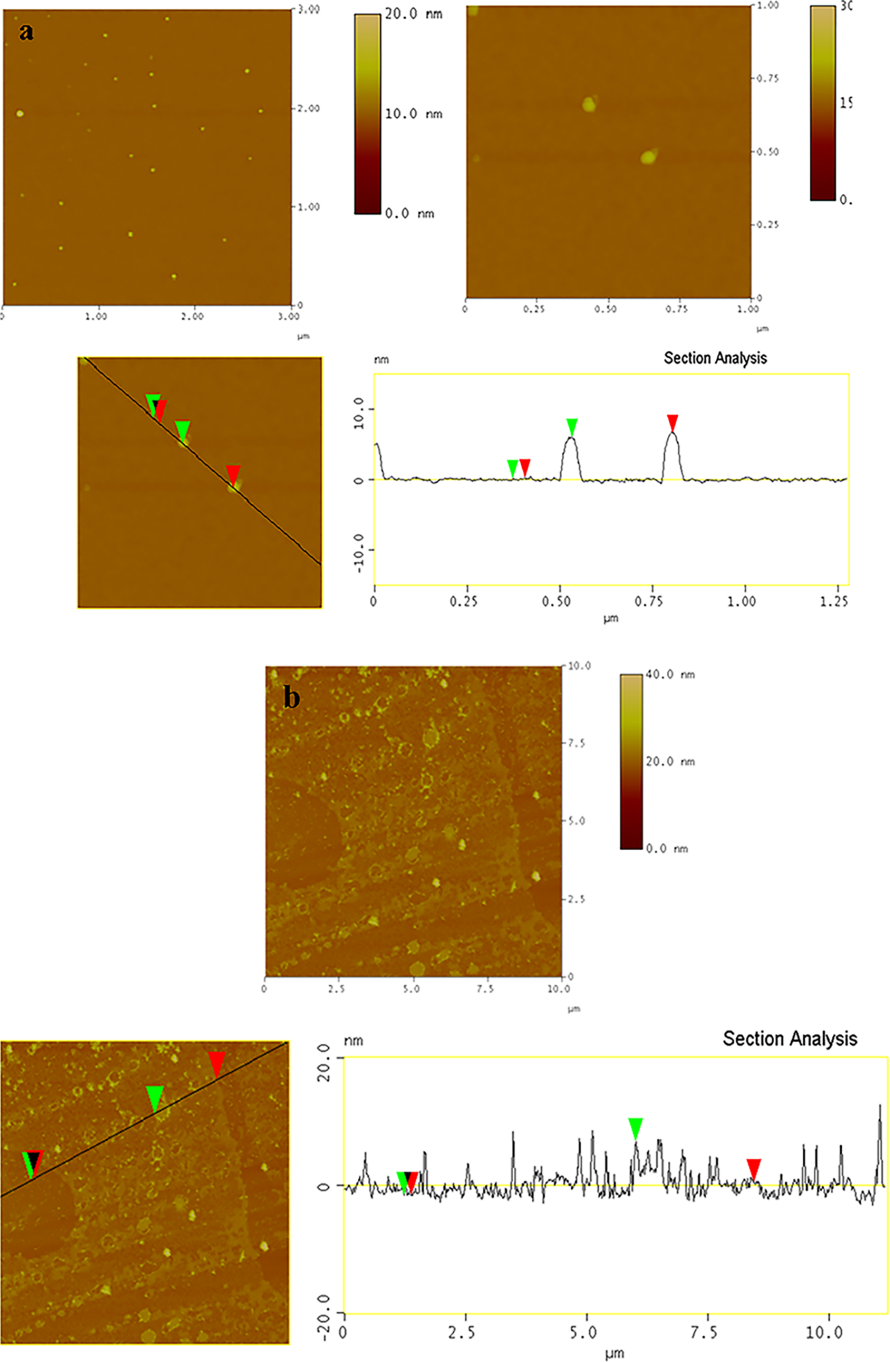
AFM height images and cross section analysis of (a) Mg–Al–NO_3_-LDHs, (b) Mg–Al-Q-LDHs, and (c) Mg–Al-Q-LDHs 65–70°.

### 3.2 Cell Viability

#### 3.2.1 Cell Viability after Exposure to Mg–Al–NO_3_-LDHs

Saos-2 and NIH/3T3 cells were exposed to Mg–Al–NO_3_-LDHs for 24 and 48 h at concentrations ranging from 10 to 1000 μg/mL. There was no evidence of toxicity in either a dose- or time-dependent manner. The survival of both cell lines was maintained at maximum levels (Figure 7a[Fig fig7],b).

**Figure 7:**
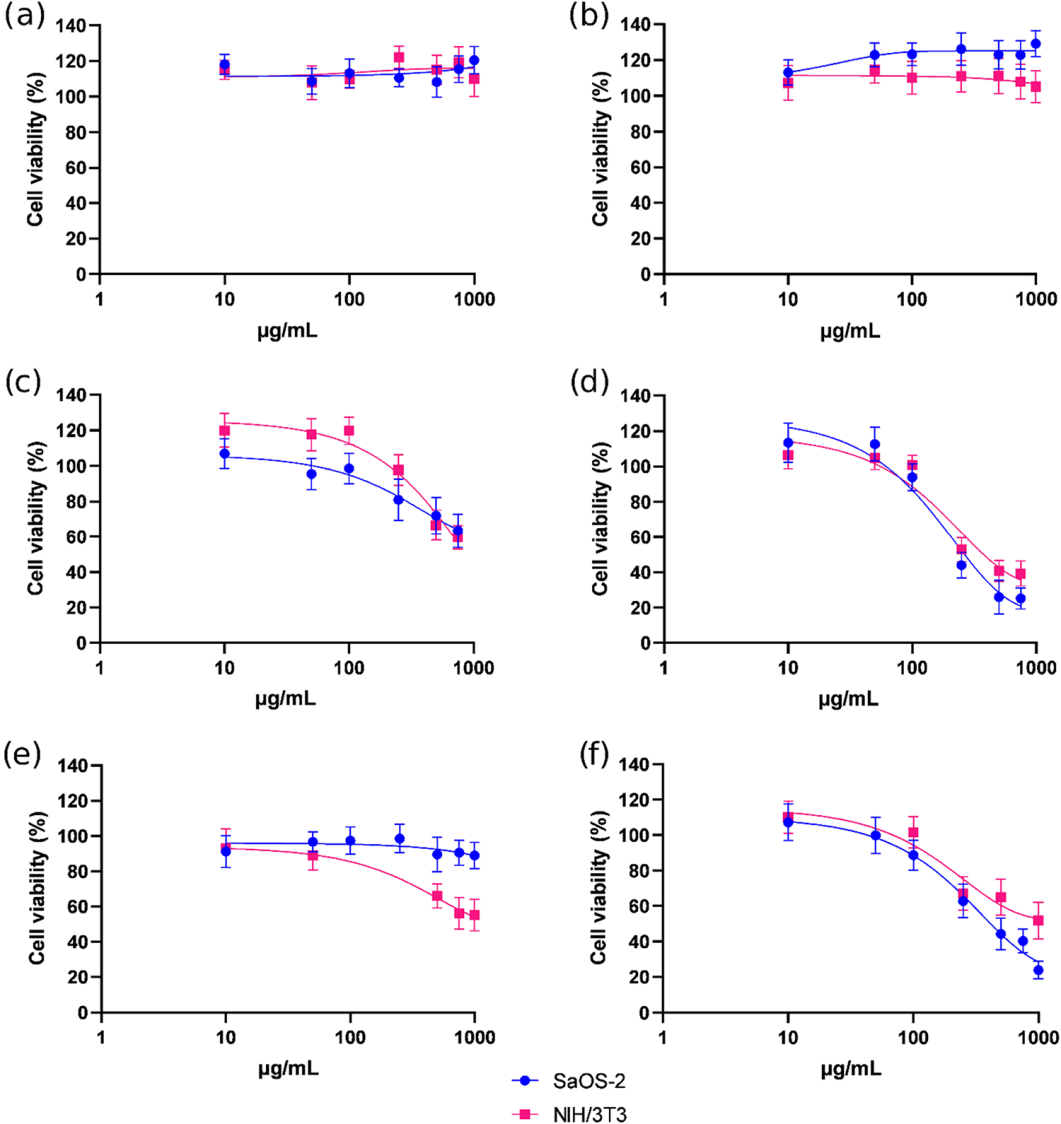
Cell viability of Saos-2 and NIH/3T3 cells after exposure to Mg–Al–NO_3_-LDHs for 24 h (a) and 48 h (b); Mg–Al-Q-LDHs for 24 h (c) and 48 h (d); and Mg–Al-Q-LDHs 65–70 °C for 24 h (e) and 48 h (f).

In line with our results, Ashtami and Mohanan conducted an MTT assay to evaluate whether 2D Zn–Al-LDH induced cytotoxic damage to human osteoblast cells (HOS). They reported that cell viability remained above 80% at the highest experimental concentration of 160 μg/mL after 48 h of exposure.^42^ Similarly, in another study, Choi et al. examined the toxicity of inorganic layered metal hydroxides on four cell lines: human lung cancer cells (A549), normal human lung cells (L-132), human cervical cancer cells (HeLa), and human osteosarcoma cells (HOS). In all four cell lines, cell viability exceeded 80% after exposure to a concentration of 500 μg/mL for 72 h.^43^ These studies support our findings, indicating that LDHs exert minimal cytotoxicity, even at high concentrations.

#### 3.2.2 Cell Viability after Exposure to Mg–Al-Q-LDHs

Saos-2 and NIH/3T3 cell lines were exposed to Mg–Al-Q-LDHs at concentrations ranging from 10 to 750 μg/mL. Mg–Al-Q-LDHs affected the cell viability of both cell lines in a similar manner (Figure 7c,d). Time- and dose-dependent effects were observed. At 24 h, at concentrations greater than 500 μg/mL, cell viability was reduced to less than 75%, and at a concentration of 750 μg/mL, it reached about 60%. At 48 h, the cytotoxic effect was greater than at 24 h. This highlights the time-dependent cytotoxic effect of Mg–Al-Q-LDHs. Cell viability was reduced to below 50% at concentrations ≥200 μg/mL, and at the maximum concentration of 750 μg/mL, it reached 25% in the Saos-2 cell line and 40% in the NIH/3T3 cell line.

Figure 7e[Fig fig7],f shows the viability of NIH/3T3 and Saos-2 cells after exposure to Mg–Al-Q-LDHs 65–70 °C for 24 and 48 h, respectively, at concentrations ranging from 10 to 1000 μg/mL. In Figure 7e, after 24 h of exposure to Mg–Al-Q-LDHs 65–70 °C, a decrease in cell viability was observed only in the NIH/3T3 cell line. At concentrations of ≥500 μg/mL, the viability was estimated below 70%, and at the maximum concentration of 1000 μg/mL, it dropped to 55%. In contrast, the viability of Saos-2 remained at ≥90%, even at a concentration of 1000 μg/mL. In Figure 7f, after 48 h of incubation with Mg–Al-Q-LDHs 65–70 °C, cell viability decreased in both cell lines. At a concentration of 250 μg/mL, viability in both cell lines dropped to below 70% At the maximum concentration of 1000 μg/mL, the viability in Saos-2 reached 23%, while in the NIH/3T3 cell line, it reached 50%. In conclusion, while the viability of Saos-2 cells was not significantly impacted by Mg–Al-Q-LDHs 65–70 °C after 24 h, it decreased substantially by 48 h. In contrast, NIH/3T3 cells showed a decline in viability starting from 24 h.

#### 3.2.3 Cell Viability after Exposure to Quercetin

Figure 8a[Fig fig8],b depicts the cell viability after exposure to quercetin for 24 and 48 h, respectively. Quercetin was added to the cells at concentrations ranging from 1 to 200 μg/mL. Cell viability was significantly reduced at concentrations greater than 25 μg/mL (Figure 8a). The cytotoxic effect of quercetin reached its maximum at 50 μg/mL in both cell lines (74% viability for Saos-2 cells and 48% for NIH/3T3 cells) and remained unchanged beyond this concentration. This indicates that the NIH/3T3 cell line is more sensitive to quercetin exposure. A similar effect was observed after 48 h of quercetin exposure, where NIH/3T3 cell viability was more significantly impacted than that of Saos-2 cells. At the higher concentration of 200 μg/mL, cell viability decreased to 50% in the Saos-2 cell line and to just 28% in the NIH/3T3 cell line.

**Figure 8:**
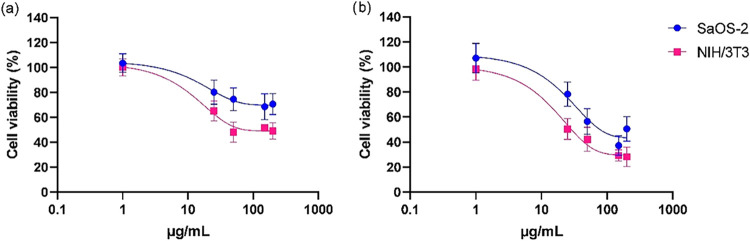
Cell viability of Saos-2 and NIH/3T3 cells after exposure to quercetin for 24 h (a) and 48 h (b).

Our findings align with the study conducted by Li et al., who reported a dose-dependent cytotoxicity of quercetin in Saos-2 and U2OS (human bone osteosarcoma epithelial cells) cell lines using the SRB technique. At the highest concentration of 100 mM, cell viability decreased to approximately 60% in the U2OS cell line and about 70% in Saos-2 cells.^44^

#### 3.2.4 Ability of Cells to Form Colonies

Figure 9(a–d) shows the ability of cells to form colonies after exposure to quercetin, Mg–Al-Q-LDHs, Mg–Al-Q-LDHs 65–70 °C, and Mg–Al–NO_3_-LDHs, respectively, at different concentrations.

**Figure 9:**
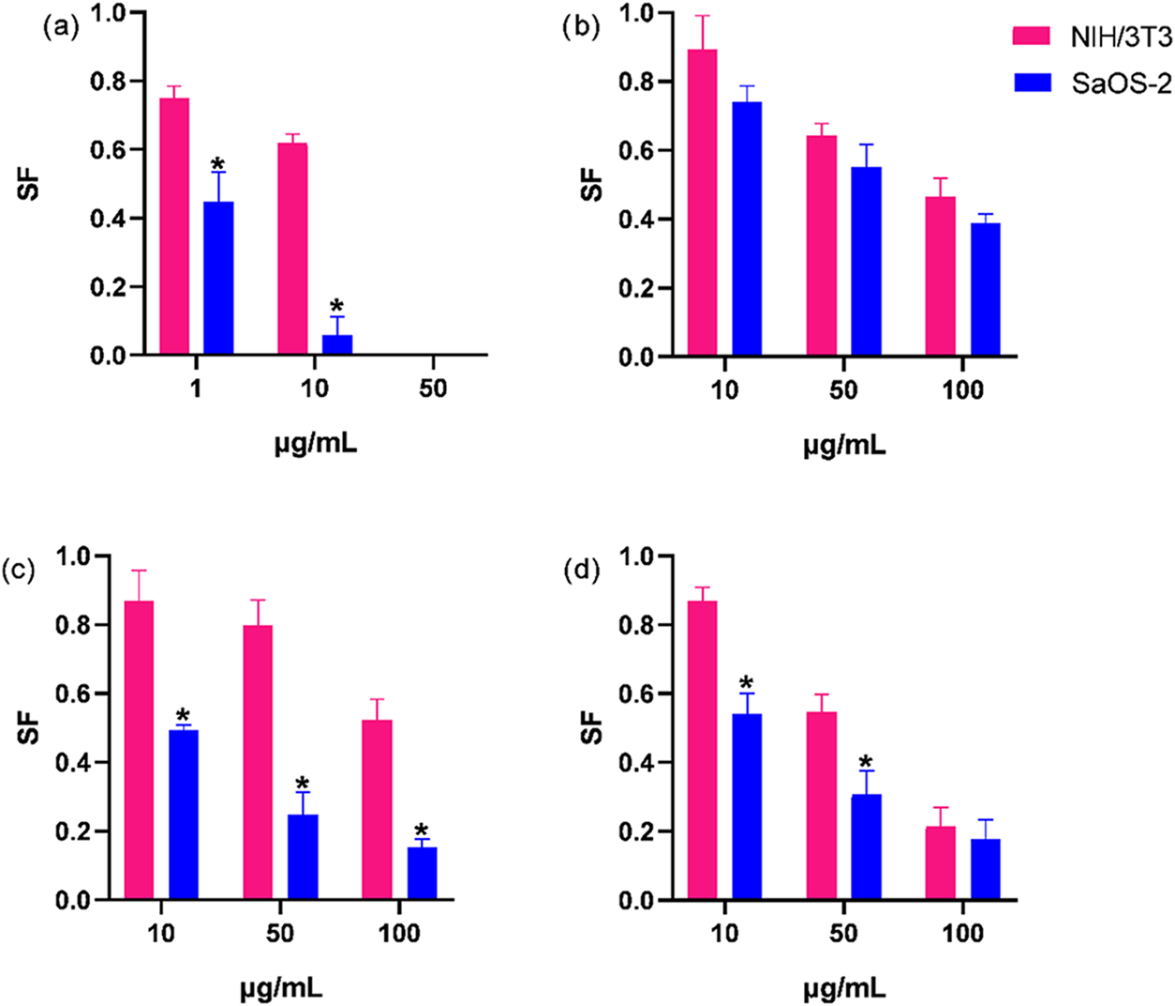
Ability of NIH/3T3 and Saos-2 cells to form colonies after a 24 h treatment with quercetin (a) at concentrations of 1,10 and 50 μg/mL, Mg–Al-Q-LDHs (b), Mg–Al-Q-LDHs 65–70 °C (c), and Mg–Al–NO_3_-LDHs (d), at concentrations of 10, 50, and 100 μg/mL*, statistically significant difference from control (*p* < 0.05).

In Figure 9a, quercetin at 50 μg/mL completely inhibited colony formation in both cell lines. At 1 and 10 μg/mL, colony formation was observed, but the survival fraction of NIH/3T3 cells decreased by 0.25 and 0.38, and that of Saos-2 cells by 0.55 and 0.95, respectively. Notably, Saos-2 cells exhibited higher sensitivity, with only 0.05 survival fraction at 10 μg/mL. Moreover, increasing the quercetin concentration from 1 to 10 μg/mL had a greater impact on the ability of Saos-2 cells to form colonies compared to NIH/3T3 cells, as the difference in their survival fraction increased from 0.3 to 0.57. This indicates that quercetin exerted less cytotoxicity on NIH/3T3 cells compared to Saos-2 cells or that NIH/3T3 cells exhibited greater resistance to quercetin treatment than Saos-2 cells, meaning they were better able to withstand the stress and continue proliferating.

In similar clonogenic assays conducted by Li et al. to evaluate the effects of quercetin on the colony-forming ability, it was revealed that quercetin treatment significantly reduced the proliferation capacity of U2OS and Saos-2 cells. Specifically, quercetin treatment reduced the colony-forming ability of U2OS cells by 68 and 84% at concentrations of 80 and 100 μM, respectively. For Saos-2 cells, the reduction was 65 and 73% at the same concentrations. These results indicate that quercetin affects the long-term capacity of bone cancer cell lines in a dose-dependent manner and are consistent with the findings of this study.^44^

Figure 9b shows colony formation after 24 h of exposure to Mg–Al-Q-LDHs at concentrations of 10, 50, and 100 μg/mL. At 10 μg/mL, NIH/3T3 cells had a 0.90 survival fraction, while Saos-2 cells had 0.75. At 100 μg/mL, both cells’ survival fraction dropped below 0.50, with concentrations ≥50 μg/mL causing irreversible damage. Figure 9c highlights that Mg–Al-Q-LDHs 65–70 °C were significantly more toxic to cancer cells. At just 10 μg/mL, Saos-2 cells showed a 0.50 reduction in colony formation, while NIH/3T3 cells were minimally affected, aligning with the goal of targeting cancer cells. Lastly, Figure 9d shows that Mg–Al–NO3-LDHs were more toxic to Saos-2 cells and equally harmful to NIH/3T3 cells at higher concentrations. Furthermore, the effect of Mg–Al-Q-LDHs synthesized at 65–70 °C (Figure 9c) compared to Mg–Al-Q-LDHs (Figure 9b) on the survival fraction of NIH/3T3 and Saos-2 cells reveals that synthesizing Mg–Al-Q-LDHs at higher temperatures can result in more toxic end products. However, the cytotoxicity of Mg–Al-Q-LDHs synthesized at 65–70 °C is more pronounced in cancer cells than in normal cells, particularly at lower concentrations (1 and 10 μg/mL).

Hae-Eun Chung et al. used the human lung alveolar carcinoma epithelial cell line (A549) to investigate colony formation following exposure to two distinct forms (carbonate and chloride) of anionic nanoclays. Their findings revealed that both forms of the anionic nanocoatings inhibited colony formation by approximately 60% at a concentration of 500 μg/mL after a 10-day incubation period.^45^ Interestingly, the A549 cell line exhibited greater resistance compared to the NIH/3T3 and Saos-2 cell lines exposed to Mg–Al–NO_3_-LDHs, where inhibition of colony formation was observed at concentrations as low as 100 μg/mL.

The cell viability results suggest that quercetin alone shows slightly greater short-term cytotoxicity (MTT assay) in NIH/3T3 cells compared to Saos-2 cells, with an average increase of 10–20% across tested concentrations. In contrast, the effects of Mg–Al-Q-LDHs are more comparable between the two cell lines. This apparent discrepancy may be attributed to differences in cellular tolerance and exposure duration, as the MTT assay suggests that NIH/3T3 cells can withstand higher doses of quercetin and Mg–Al-Q-LDHs during prolonged exposure (48 h). Importantly, the clonogenic assay demonstrates that both quercetin and Mg–Al-Q-LDHs induce irreversible damage primarily in cancerous Saos-2 cells, which fail to maintain long-term viability. These observations highlight that while in short-term exposure, normal cells show slightly higher sensitivity, the overall long-term cytotoxic effect is selective for cancerous cells.

#### 3.2.5 Determination of Reactive Oxygen Species Formation

In the NIH/3T3 cell line, 10 μg/mL Mg–Al–NO_3_-LDHs and Mg–Al-Q-LDHs reduced intracellular ROS by 15 and 17%, respectively (Figure 10a). In the Saos-2 cell line, Mg–Al–NO_3_-LDHs had no effect on ROS, while Mg–Al-Q-LDHs reduced ROS by 42% (Figure 10b). Notably, Mg–Al-Q-LDHs (65–70 °C) and quercetin had opposite effects on the ROS in the two cell lines. In NIH/3T3 cells, they increased ROS (Quercetin ∼30%, Mg–Al-Q-LDHs 65–70 °C ∼12%), whereas in Saos-2 cells, they reduced ROS (Quercetin ∼27%, Mg–Al-Q-LDHs 65–70 °C ∼25%). The profound differentiation in intracellular ROS detection seen in NIH/3T3 and Saos-2 cells could be a result of the significant differences between cancer and normal cells in their uptake mechanisms. Especially, the presence of quercetin (in Mg–Al-Q-LDH, Mg–Al-Q-LDHs synthesized at 65–70 °C, as well as quercetin alone) plays an important role in cancer cells. This is likely due to their distinct metabolic requirements, surface receptor profiles, and microenvironment adaptations, which lead to higher uptake. This, in turn, disrupts their redox equilibrium and, as observed in the clonogenic assay experiments, results in a loss of their ability to maintain normal proliferation rates.

**Figure 10:**
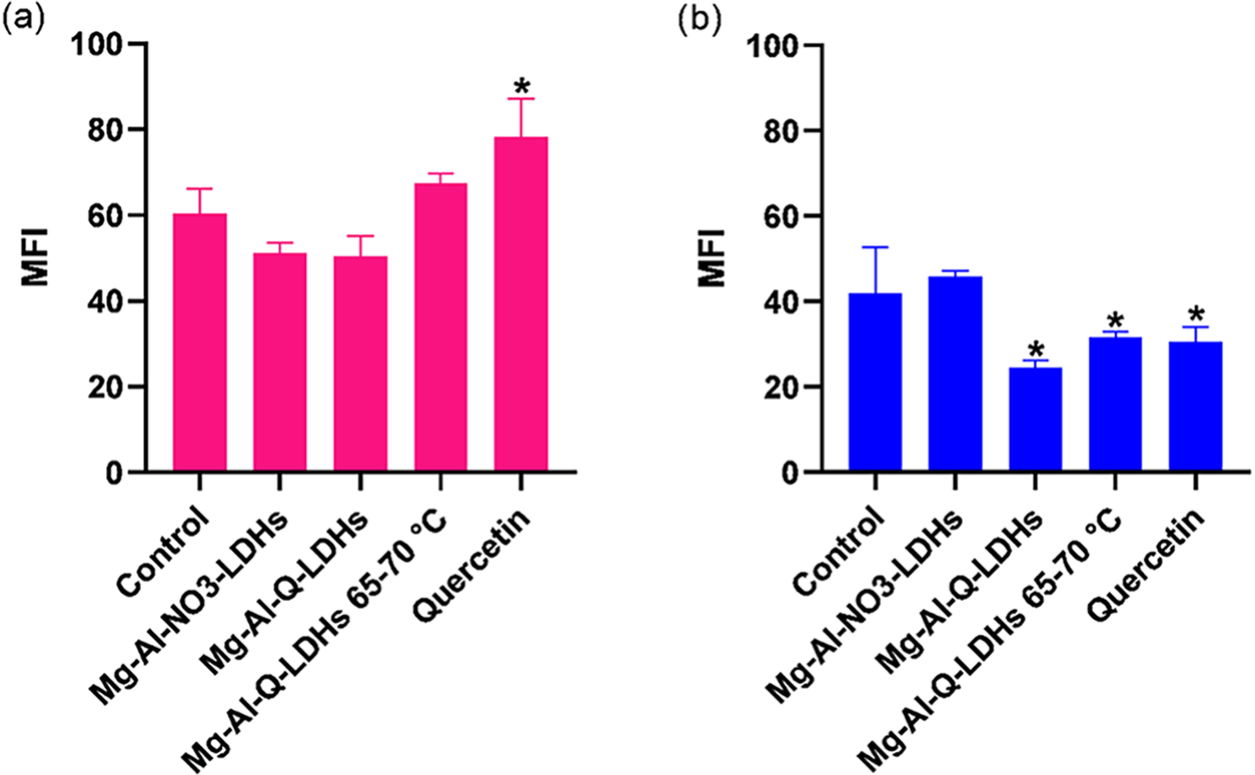
Reactive oxygen species production in NIH/3T3 (a) and Saos-2 (b) cell lines after 24 h incubation with quercetin, Mg–Al-Q-LDHs, Mg–Al-Q-LDHs 65–70 °C, and Mg–Al–NO3-LDHs at the concentration of 10 μg/mL*, statistically significant difference from control (*p* < 0.05).

Similar experiments in the literature have used the polyphenols morin and fisetin to study the production of ROS in NIH/3T3 cells. Unlike quercetin in this study, these polyphenols did not promote ROS production. It should be noted, however, that the concentration of quercetin used was approximately three times higher than that of fisetin and morin.^46^ In contrast to a study where curcumin increased ROS production in Saos-2 cells, the present study found that quercetin reduced ROS production in the Saos-2 cell line.^47^ These results show that different polyphenols have unique effects on cells and the concentration of exposure plays a crucial role in determining these outcomes. Soo-Jin Cho et al. found that layered double hydroxide nanoparticles (LDH-NPs) generated ROS in A549 cells when exposed to concentrations of 250–1000 μg/mL for 24 h. These results, along with our findings, indicate that LDH nanoparticles do not produce ROS in cells.^48^

#### 3.2.6 Saos-2 Cells Labeling Study

Exposure of Saos-2 cells to low concentrations (0.5 and 1.0 μg/mL) of DAPI-labeled LDH revealed that LDH nanoflakes were able to cross the cytoplasmic membrane and enter the cells (Figure 11). This internalization is likely mediated by electrostatic interactions between the positively charged LDHs and the negatively charged cell membrane, followed by uptake through endocytosis. Previous studies on Saos-2 cells have similarly demonstrated that methotrexate-LDH conjugates exhibited enhanced cellular penetration and sustained intracellular drug retention compared with the free drug, findings attributed to clathrin-mediated endocytotic uptake of LDH-drug complexes.^49^

**Figure 11:**
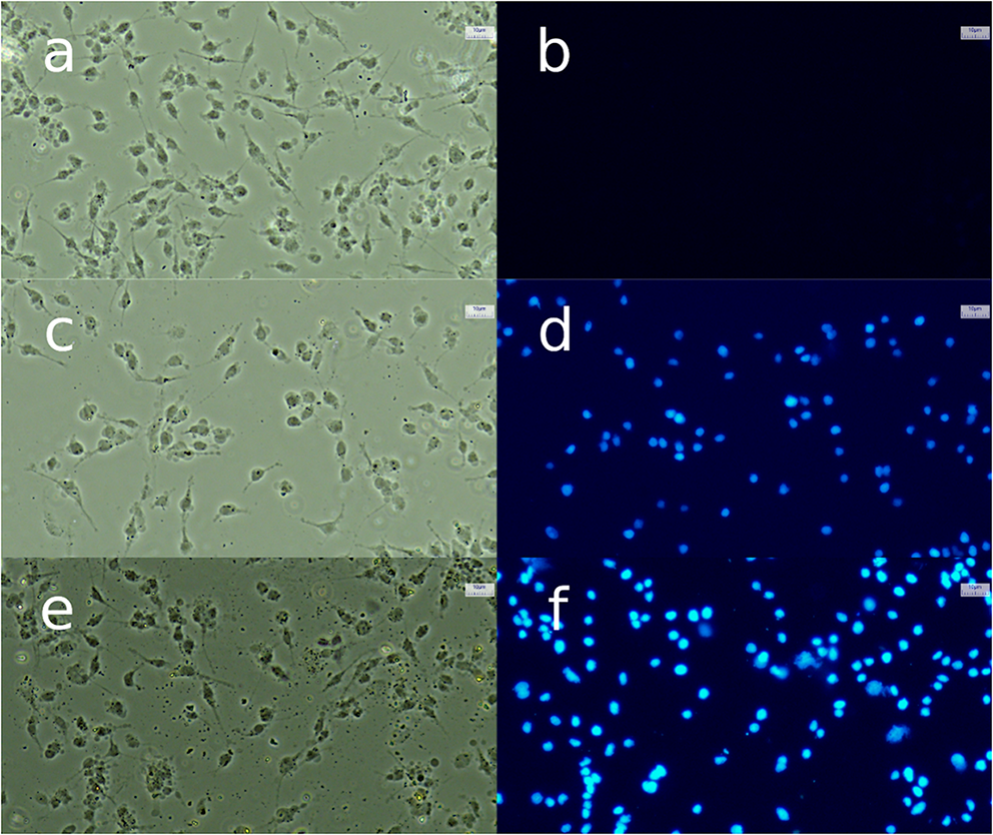
Bright-field (a–e) and fluorescence (b–f) microscopy images showing uptake of DAPI-labeled LDH by Saos-2 cells. (a, b) Control cells; (c, d) cells treated with 0.5 μg/mL LDH nanosheets; and (e, f) cells treated with 1.0 μg/mL LDH (scale bar, 10 μm).

## 4 Conclusions

In summary, this study successfully demonstrated the synthesis and characterization of Mg–Al–NO_3_-LDHs and their quercetin-intercalated counterparts, Mg–Al-Q-LDHs, through various analytical techniques, including FTIR, Raman spectroscopy, X-ray diffraction, and thermogravimetric analysis. Cytotoxicity studies reveal that Mg–Al–NO_3_-LDHs exhibited minimal toxicity across both Saos-2 and NIH/3T3 cell lines, maintaining high cell viability even at elevated concentrations. Conversely, the quercetin-intercalated LDHs showed a significant time- and dose-dependent reduction in cell viability, particularly affecting Saos-2 cells. This indicates that although quercetin enhances the cytotoxic properties of LDHs, the effects vary across different cell lines, emphasizing the potential of Mg–Al-Q-LDHs for targeted cancer therapies. Moreover, the study highlights the complex interplay between quercetin and ROS production, revealing differing effects depending on the cell type and exposure concentration. These findings contribute to the understanding of LDH-based nanomaterials in biomedical applications, suggesting that the careful design of intercalated compounds can enhance therapeutic efficacy while minimizing toxicity. Overall, the results underscore the potential of Mg–Al-Q-LDHs as promising candidates for further development in drug delivery systems and cancer treatment strategies.

## Data Availability

Data will be made available upon request.

## Abbreviations

LDHs: Layered Double Hydroxides
Q: Quercetin
FT-IR: Fourier Transform Infrared Spectroscopy
AFM: Atomic Force Microscopy
TGA: Thermogravimetric analysis
XRD: X-ray diffraction
ROS: Reactive Oxygen Species
DMEM: Dulbecco’s Modified Eagle’s Medium
FBS: Fetal Bovine Serum
DMSO: Dimethyl sulfoxide
MTT: 3-(4,5-dimethylthiazol-2-yl)-2,5-diphenyltetrazolium bromide
CFU: Colony-forming unit
HBSS: Hanks’ Balanced Salt Solution
DCFDA: 2′,7′-dichlorodihydrofluorescein diacetate
PI: Propidium Iodide
ROS: Reactive Oxygen Species
NSAIDs: anti-inflammatory drugs
PBS: phosphate buffered saline
SPSS: Superior Performance Software System
